# Effectiveness of telehealth in preventive care: a study protocol for a randomised controlled trial of tele-exercise programme involving older people with possible sarcopenia or at risk of fall

**DOI:** 10.1186/s12877-023-04535-4

**Published:** 2023-12-13

**Authors:** Karly O. W. Chan, Peter P. Yuen, Ben Y. F. Fong, Vincent T. S. Law, Fowie S. F. Ng, Wilson C. P. Fung, Tommy K. C. Ng, I. S. Cheung

**Affiliations:** 1 College of Professional and Continuing Education, The Hong Kong Polytechnic University, PolyU Hung Hom Bay Campus, 8 Hung Lok Road, Hung Hom, Kowloon, Hong Kong SAR China; 2 School of Management, Tung Wah College, Kowloon, Hong Kong SAR China; 3 Hong Kong Telemedicine Association, Hong Kong, Hong Kong SAR China

**Keywords:** Tele-exercise, Falls, Older people, Exercise adherence

## Abstract

**Background:**

Continuous loss of muscle mass and strength are the consequences of the ageing process, which increase the risk of falls among older people. Falls can lead to severe consequences such as bone fractures and hampered physical and psychological well-being. Regular exercise is the key to reversing muscle atrophy and relieving sarcopenia. However, the frailty of older people and the recent COVID-19 pandemic may affect their confidence to leave home to attend classes in the community. A feasible and effective alternative should be explored.

**Methods:**

The primary objective is to evaluate the effectiveness of tele-exercise (TE) in relation to physical functioning and exercise adherence among community-dwelling older people at risk of falls in comparison with a community-based group (CB). The secondary objective includes evaluating older people’s experience with tele-exercise, emphasizing their psychological welfare, social well-being, and acceptance of the telehealth approach. The design, conduct, and report follow the SPIRIT guidelines (Standard Protocol Items: recommended items to address in a Clinical Trial Protocol and Related Documents). Older people will be recruited from 10 local community centres in Hong Kong and randomly allocated into two groups. All participants will attend the exercise training 3 days per week for 3 months but the mode of delivery will differ, either online as the tele-exercise group (TE) or face-to-face as the community-based group (CB). The outcome measures include muscle strength, physical function, exercise adherence and dropout rate, psychological and social well-being will be assessed at the baseline, and the 3rd, 6th and 12th month. Some participants will be invited to attend focus group interviews to evaluate their overall experience of the tele-exercise training.

**Discussion:**

Tele-exercise reduces the barriers to exercise, such as time constraints, inaccessibility to facilities, and the fear of frail older people leaving their homes. Promoting an online home-based exercise programme for older people can encourage them to engage in regular physical activity and increase their exercise adherence even when remaining at home. The use of telehealth can potentially result in savings in cost and time. The final findings will provide insights on delivering exercise via telehealth to older people and propose an exercise delivery and maintenance model for future practice.

**Trial registration:**

Chinese Clinical Trial Registry (https://www.chictr.org.cn/hvshowprojectEN.html?id=219002&v=1.1), registration number: ChiCTR2200063370. Registered on 5 September 2022.

## Background

The decline of muscle mass and strength with age will expose older people to a higher risk of sarcopenia and falls. Falls are common among older people. The 1-year prevalence of falls among community-living older people aged 65 years or above with at least one fall is 20% with the fall rate (number of falls per 100 people) 29% in Hong Kong [1]. After the age of 75, the fall rates are even higher [2]. Hip fracture is one of the outcomes of falls among older people and is associated with rehospitalization and increases in hospital stay and mortality rates [3, 4]. Sarcopenia is one of the main reasons of disability, falls and hip fractures in older people and it is a challenge in regard to healthy ageing [5]. To reduce the prevalence and incidence of falls and sarcopenia among older people, exercise training is found to have positive effects on balance, muscle mass and strength, as well as physical functioning [6, 7]. Nevertheless, older people are less likely to participate in fall prevention programmes with exercise classes in Hong Kong because frail older people may not be confident to leave their home and join a programme in the community [8]. Thus, it is necessary to find feasible alternatives to encourage and facilitate older people to develop regular exercise habits.

Telehealth refers to the remote delivery of health care services such as clinic appointments and exercise training (tele-exercise), using information and communication technologies (ICT) where the provider and service recipient are in different physical locations [9]. Occupational and physical therapy practitioners increasingly use tele-exercise to enable real-time interactions between instructors and community-dwelling older people in order to save money and waiting time [10, 11]. Promoting an online home-based exercise programme for older people can encourage them to engage in regular physical activity and increase their exercise adherence even when remaining at home. Tele-exercise was particularly important when people needed to adhere to social distancing during the pandemic.

The Otago Exercise Programme (OEP) is an evidence-based fall prevention programme developed in the late 1990s [12, 13]. It consists of five warm-up exercises (head movement, neck movement, back extension, trunk movement and ankle movement) and 17 strength and balance exercises. The original programme was designed as a home-based exercise for older people with guidance and monitoring provided by trained professionals [14]. Many studies have implemented the OEP as an intervention programme for older people to prevent falls and demonstrated positive outcomes, including reduced fall-related injuries and improved functional mobility and balance [15–21]. Similar results were observed in a modified OEP too. The modified formats included additional multisensory balance exercises, augmented reality, training in groups and a DVD delivery format. The OEP in a group is preferable as exercising in a class can offer additional psychological and social benefits [22]. In addition, engagement between the participants and instructors is an essential factor for exercise maintenance, as demonstrated by Davis et al. [23] who delivered the OEP by video along with a single visit by a physical therapist. Compared with studies that involve more participant interaction, the OEP group with less participant interaction resulted in a higher rate of loss to follow-up, with approximately 29.5% of participants failing to complete the post-intervention assessment. Thomas et al. [24] believe that maximizing compliance can improve health outcomes. The OEP is more suitable for older adults who are more prone to falls and less active as the movements required in the exercises are straightforward and of low intensity. However, it lacks interesting variations of movements that may affect compliance [25]. The number of dropouts of OEP modified formats reached 30% [22].

Considering the possible benefits of tele-exercise, as well as the factors that affect exercise compliance and exercise maintenance, we hypothesize that the modified Otago exercise programme delivered via the tele-exercise approach is as effective as traditional exercise programmes among older people at risk of falls. The purposes of this study are to (i) evaluate the effectiveness of tele-exercise on physical functioning and exercise adherence among community-dwelling older people with possible sarcopenia or at risk of falls in comparison with a community-based group, (ii) to evaluate older people’s experience with tele-exercise, with an emphasis on their psychological welfare, social well-being, and acceptance of the telehealth approach.

## Methods/design

**Fig. 1:**
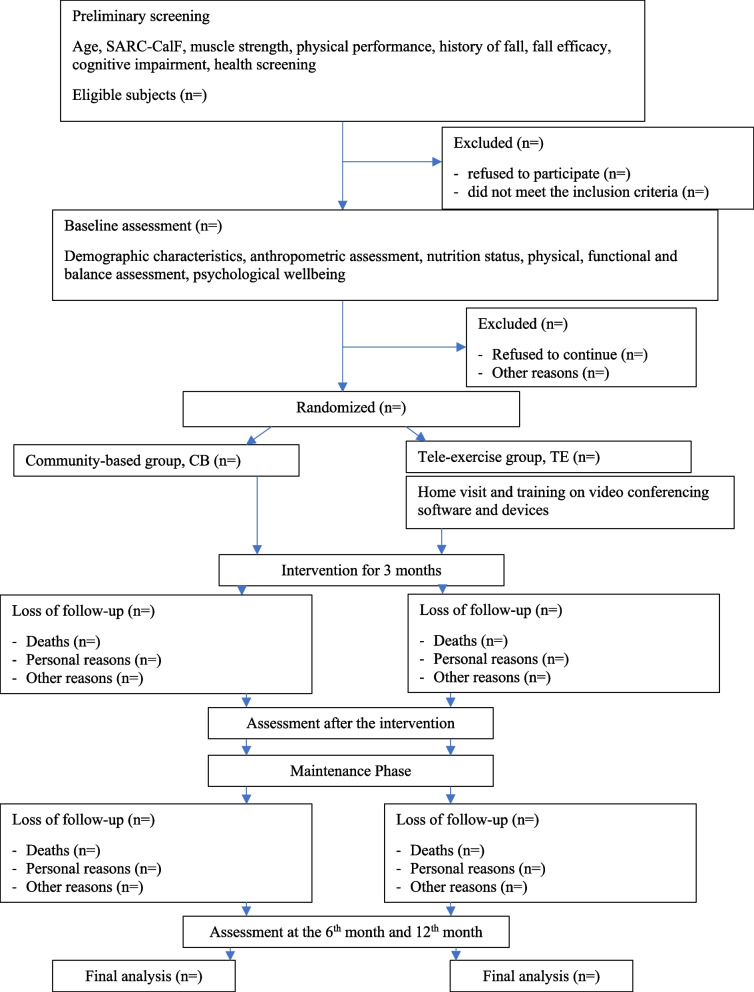
Flow chart of the study

**Fig. 2:**
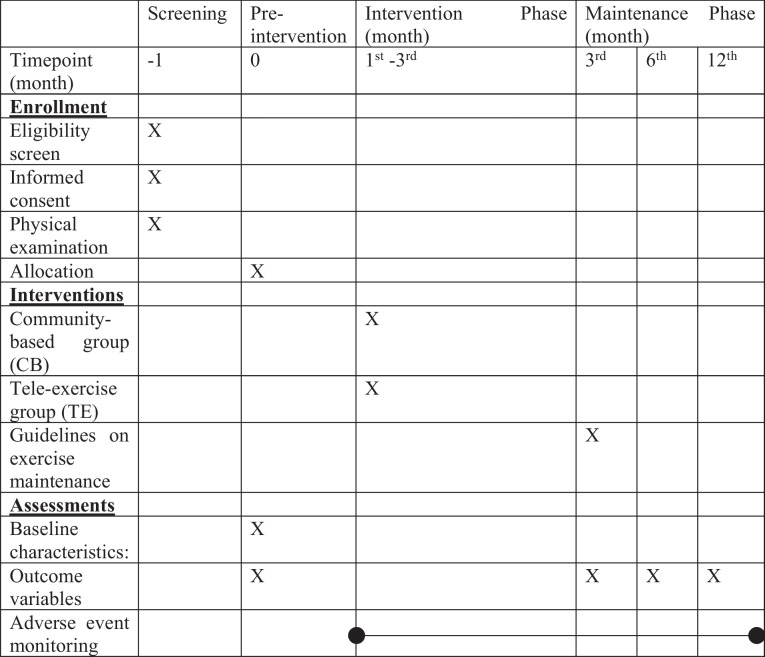
Schedule of enrollment, interventions, and assessments based on SPIRIT2013 statement [26]

 This is a 3-month single-blind, randomized controlled trial (RCT) with subsequent 9 -month follow-up comparing online (TE) with face-to-face exercise training (CB) for community-dwelling older people at risk of falls. The design and conduct of the study follows the requirements of the Standard Protocol items: Recommendations for Interventional Trials (SPIRIT) checklist [26], as shown in the flow chart of the study in Fig. 1. The protocol reported is based on the SPIRIT 2013 statement [26] (Fig. 2). The participants will be recruited from elderly community centres in Hong Kong. To facilitate the recruitment of participants, health talks about fall prevention and maintaining muscle mass will be arranged at local community centres. The potential participants will be screened and assessed through on-site enquiries and video interviews.

### Ethical and legal aspects

This study protocol was approved by the Ethics Committee of the College of Professional and Continuing Education (RC/ETH/H/0029) and was registered in the Chinese Clinical Trial Registry (ChiCTR2200063370) on 5 September 2022. All participants will be asked to agree and sign an informed consent form to participate in the study.

### Eligibility criteria

The target group of this research is the community-dwelling older people with possible sarcopenia or at risk of falls in Hong Kong. The inclusion criteria and the exclusion criteria are listed in Table 1. The diagnosis of possible sarcopenia is based on the recommendation of the Asian Working Group for Sarcopenia 2019 (AWGS) [27] to assess the SARC-CalF scores, muscle strength and physical performance. The perceived fear of falls is measured by the Chinese version of Fall Efficacy Scale-International (FES-I). The Mini-Mental State Examination (MMSE) determines if participants show signs of cognitive impairment. Medical clearance will be performed according to the guidelines of the American College of Sports Medicine (ACSM).

**Table 1:** Inclusion and exclusion criteria

Inclusion criteria	Exclusion criteria
• permanent residents of Hong Kong • aged 65 years or above living independently at home • diagnosed with possible sarcopenia and/or having had either a fall in the past year or fear of falling with a score of ≥ 20 points on FES-I • able to speak and listen to Cantonese • able to walk without mobility aids in their home	• diagnosed with dementia or cognitive impairment, Mini-Mental State (MMSE) < 25 • severe vision impairments that do not permit the use of a tablet, reading of the exercise booklet, and completing the activity diaries • unable to pass the preparticipation Health Screening of the ACSM and the subsequent medical clearance • regular physical activities in the past 6 months based on the definition of WHO [28]

### Sample size calculation

In Hong Kong, there are 1,322,000 residents who are aged 65 years or above [29] and it is estimated that 118,980 of them suffer from sarcopenia (prevalence is 9% for those aged 65 or above [30, 31] and 264,400 of them have had at least one fall in the past year [1]. Calculations were performed using G*Power software and an assumed effect size of 0.367 based on a previous study by Benavent-Caballer et al. [32] on the Time Up and Go Test. Considering a 95% confidence level, a power of 80%, and 20% attrition rate (including mortality) [33], a sample size of at least 92 are required.

### Recruitment

The potential participants will be recruited from ten community centres in Hong Kong. They will receive information about this study through a series of health talks and posters displayed in their community centres. Interested participants will register in the community centres. Our project assistant will contact them to assess their eligibility according to the inclusion and exclusion criteria and explain the details of the study to them. Eligible participants will sign the informed consent form voluntarily. Recruitment started in September 2022 and will end when 92 participants have been enrolled.

### Randomization and blinding

After the screening phase, ten community centres will be coded and then randomized such that a similar number of centres will be allocated to either the tele-exercise group (TE) or community-based group (CB) in a concealed, blinded fashion to minimize cross-over contamination between the groups. An independent assistant will oversee the randomization procedures and allocation sequence. Around ten participants will be recruited from each centre. The assessments will be performed by trained outcome assessors who will be blinded to the group assignments. It is impossible to blind the participants in the training mode. However, they cannot communicate with other groups of participants because they are recruited from different locations. The instructors and researchers will not be blinded and will be asked to refrain from discussing the group assignments of the participants with the outcome assessors.

### Baseline assessment

The details of the study will be explained to all participants, who will sign the consent form. Assessment will be performed before the intervention period, at the end of the 3rd, 6th and 12th months. The baseline assessments include the Chinese version of Fall Efficacy Scale-International (FES-I), fall incidence, SARC- CalF scoring, right-hand and left-hand grip strength, Timed Up and Go test, Berg Balance Scale, 6-metre walk test, Functional Reach Test, Physical Activity Scale for the Elderly (PASE) [34], Daily activity level, Rosenberg Self-esteem Scale and EQ-5D-3 L. The weight, body fat and skeletal muscle mass will be measured by a bioimpedance scale.

### Interventions

All participants will attend the exercise training 3 days per week for 3 months, under the delivery mode of either tele-exercise or a community-based approach. The community-based group will serve as a positive control as the effectiveness of the OEP to prevent falls is well established [24] and the aims of the current study focus on the effectiveness of the new mode of delivery, i.e. tele-exercise. The exercise programme will be structured with a warm-up, Otago strengthening and balance retraining exercises, “workouts on-demand” and cool down (Table 2). The OEP consists of five warm-up exercises (head movement, neck movement, back extension, trunk movement and ankle movement) and 17 strength and balance exercises [14]. For the “workouts on-demand” part, participants will express their needs such as focusing more on the upper body, flexibility, or strength. The instructor will present real-time demonstrations to promote exercise empowerment. Both groups of participants will be divided into classes of 9–10 people. In addition, they will be asked to maintain their daily number of steps at 10,000 or increase their step count by 3,500 compared with their usual habit. They will be required to record all the exercises they perform, the number of steps achieved and the fall incidence during the 12 months.

**Table 2:** Lesson plan for both TE and CB groups

Content	Duration
Warm-up (dynamic and static stretching)	~ 10 min
A short pause to allow participants to be ready for the next part and interact with the instructor	
Otago exercise programme-strengthening exercises	~ 10 min
A short pause to allow participants to be ready for the next part and interact with the instructor	
Otago exercise programme- balance retraining exercises	~ 10 min
Workouts on-demand allow participants to - express their opinions on the exercises - request targeted training for specific body parts at a higher or lower level	5–10 min
Cool down	~ 10 min

#### Intervention-tele-exercise group

For the TE group, the exercise programme will be delivered via Zoom (Zoom Video Communications Inc.), which allows online communication with participants, screen sharing, and video recording. To run the exercise training smoothly, participants will be given a tablet computer with a stand if they do not have suitable devices. Those who do not have a Wi-Fi connection will be provided with data sim cards that allow internet access. Home visits will be arranged before the intervention to support the participants technically in using and setting up online communication applications in their homes. The position of the tablet, the chair and the participant for the best visual effect and interaction will be marked and recorded. A sturdy chair will act as a nearby support for the participant during training. The participants will be reminded to turn on their tablet and the Wi-Fi connection before the class so that the project team will control the tablet remotely and solve most technical problems immediately. Participants will attend the first lesson in the community centre to familiarise themselves with the exercise safety rules and the OEP. After that, all the training sessions will be delivered online. To enhance the interaction, the participants will be required to turn on their camera and microphone. The team will monitor the progress of the participants regularly.

#### Safety measures for the tele-exercise group

The team will conduct a home visit to every participant of the TE group to ensure exercise safety and offer technical support. The setup of the tablet, as well as the home environment of the participants will be checked at the beginning of every lesson. The participants will be asked to perform all the exercises of the OEP under the supervision of the instructor during the first lesson in the community centre. All participants will start the exercises at the beginner level (level A) for which they will be asked to hold the support for all the standing and walking exercises. Among the Otago exercises, backward walking may require more attention. The team will also estimate the space required for walking and the upper body exercise for the “Workouts on-demand” session. If 4 sets of 10 steps cannot be performed due to limited space, the participant will be asked to do 10 sets of 4 steps instead.

**Fig. 3:**
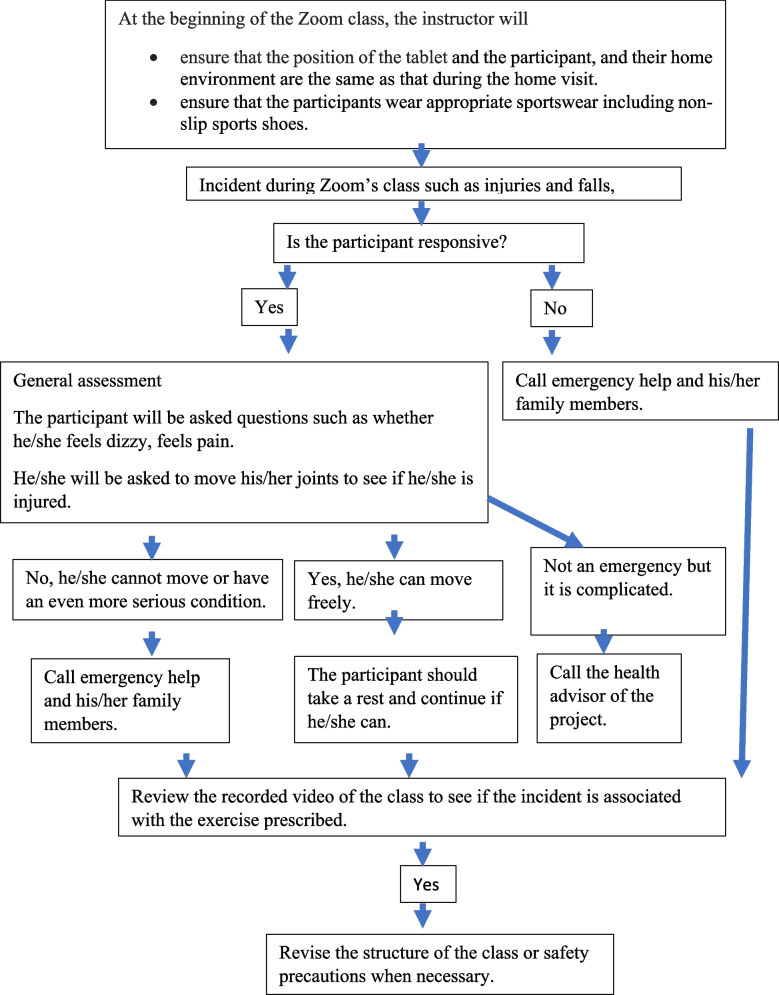
Sample safety protocol

 In addition, the participants will be reminded to wear appropriate exercise gear including non-slip sport shoes as well as stay hydrated. If the participants cannot follow the Zoom training properly, another home visit will be conducted. If an incident such as a fall occurs, we will endeavour to communicate with the participant to ascertain if he or she is responsive and understand the seriousness of the incident. If the participant falls unconscious, we will call for emergency help and contact his or her relatives immediately. A safety protocol has been developed to guide the emergency procedures (Fig. 3).

#### Intervention-community-based group

The team will organize classes in the community centres. The location of the course will be based on the residence of the participants being recruited. The instructor will deliver the OEP to the participants face to face. Like the TE group, the instructor will regularly monitor and review their progress.

#### Intrinsic differences between tele-exercise and community-based group

There are intrinsic differences in the delivery mode between CB and TE. In CB, participants have direct communication with the instructor and their classmates. The instructor can correct the movements of participants verbally and physically. In contrast, in TE, the instructor can observe all the participants in real time as their images are projected onto a large screen (Diagonal: 100 inch). The instructor can only correct the movement of the participants verbally but not physically (Table 3). On the other hand, we will pin the image of the instructor to the screen of all the participants. Participants can only observe the movements of the instructors on their screens and not those of their peers.

**Table 3:** Intrinsic differences between TE and CB

	TE	CB
Mode of delivery	Online via Zoom	Face-to-face
Location of the class	Participants’ home	Community centre
Interaction		
(i) verbally between the instructor and participants	Directly with minimal delay	Directly
(ii) verbally between the participants	Possible after class	Possible during and after class

#### Maintenance phase

After 3 months of training, a workshop will be arranged to remind the participants to maintain the exercise habit and achieve a daily step count of > 10,000 steps or increase their step count by 3,500 every three months until they reach 10,000 steps per day. The exercise booklets of our exercise programme will be distributed to all participants. The exercise videos will be sent to them via WhatsApp, or presented to them in a way that they can be easily accessed. They will be reminded by phone at least twice per month to record their exercise and step count in the diary provided.

#### Strategies to enhance the exercise experience of the participants

##### Self-efficacy 

Every participant will have an exercise booklet, pedometer, and activity diary to remind them to be active daily. For the TE group, the assistants will conduct home visits to help the participants to set up their tablets and inspect their home environment. Colourful notecards will be provided to remind the participants of the procedures for attending the online class. Exercise videos will be uploaded to social media so that the participants can follow them during the maintenance phase.

##### Self-confidence

All classes of TE and CB will be led by the same instructor, aged 66, who is experienced in teaching older people. He understands the needs of older people and acts as a role model for the participants. The class size will be between 9 and 10 for both TE and CB, which can promote better interaction and allow the instructor to become familiar with all of them. The first week will allow the participants to get used to the movements. The instructor will correct their movement, assess every participant’s performance and encourage them to complete the exercise or progress to a higher level. The level of exercise will be tailored for all the participants so that they will gain confidence in the completion of the prescribed exercises. The instructor will commend the participants for their effort in performing the prescribed exercises regularly during each lesson. Our team will encourage the participants to be active during the intervention and maintenance phases through regular phone calls or text messages. We will set up an easily achievable target for the participants such as walking 500 steps more for the following week.

##### Empowerment 

Ten minutes will be reserved in each lesson for the participants to express their comments on the exercises and request the exercises that they want. They can discuss with the instructor their performance and whether they can proceed to the next level. The research team will organize a maintenance workshop to present the exercise guidelines, exercise booklet and videos for all the participants to maintain their exercise habits after the 3-month exercise classes. Some reliable web resources will also be recommended to the participants. They will also be advised to participate in exercise courses provided by other community centres.

### Outcome measures

#### Primary outcomes

Trained and blinded assessors will conduct all outcome assessments.

The primary outcomes are the change in muscular strength and physical function among older people compared with different time points, i.e. at the baseline, and after 3, 6 and 12 months. The outcomes include risk of falls (Chinese version of Fall Efficacy Scale-International) and fall incidence, right-hand and left-hand grip strength, 6-meter walk test, SARC-CalF, Timed Up and Go test (TUG), Berg Balance Scale (BBS), functional reach test, skeletal muscle mass and fat mass.

- Chinese version of Fall Efficacy Scale-International: This is a validated measurement of fall efficacy with 16 questions regarding confidence of fall prevention at different situations on a four-point scale ranging from “not at all concerned” to “very concerned”. The score ranges from 16 to 64 and a lower score indicates a low fear of falling [35]. A perceived fear of falling is defined as points ≥ 20 [36].
- History of falls and fall incidence: A fall is defined as “an unexpected event in which the participant comes to rest on the ground, floor, or lower level, with or without injury” [37]. The history of falls including number, circumstance and severity in the previous 12 months will be assessed at the baseline [17]. The participants will be required to record their fall incidence prospectively in their diaries. The project assistant will also contact the participants regularly to ensure that they record the correct information.
- Right-hand and left-hand grip strength: This measurement will be performed by using a hand dynamometer (Takei Scientific Instruments Co. Ltd, Tokyo, Japan). The participants will be instructed to stand with their elbow extended and squeeze the dynamometer as hard as they can. Three attempts for both hands will be allowed and the mean of three scores will be counted [16].
- 6-metre walk test: This is the test to assess the gait speed of the participants. The participants will be asked to walk as fast as possible without running for 10 m, which comprises a 2-metre approach, 6 m for time measurement and 2 m beyond the measurement. The units of this measurement are in seconds and will be divided by 6 to calculate the gait speed in m/s. Two attempts will be allowed and the mean of two scores will be counted [17].
- SARC-CalF: This is used to screen sarcopenia. It consists of six components namely strength, assistance in walking, rising from a chair, climbing stairs, falls and calf circumference [38, 39]. According to the Asian Working Group for Sarcopenia 2019 [27], diagnosis of possible sarcopenia includes SARC-CalF ≥ 11, handgrip strength (M:<28 kg, F < 18 kg) and 6-metre walk test < 1.0 m/s.
- Timed Up and Go Test: This is a test of functional lower-limb mobility. The participants will be seated with arms resting on the armrests. They will be asked to walk for 3 m, return to the chair, and sit down again. The time will be counted from standing to sitting back on the chair. Three attempts will be allowed and the mean of three scores will be counted [16].
- Berg Balance Scale score: This is a valid and reliable scale consisting of 14 static and dynamic balance activities. The score of each activity ranges from 0 to 4 and the total score ranges from 0 to 56 [40].
- Functional Reach Test: This test is used to assess dynamic bilateral stance balance. A metre ruler is fixed on the wall at participants’ shoulder height and the participants will be asked to stand next to but not touching the wall. The arm near the wall needs to be raised at 90° flexion and the third finger will be the initial position along the metre ruler. The participants will be asked to reach as far as possible without taking a step, bending their knees or falling over. The end position on the ruler will be marked. Three attempts will be allowed and the mean of three scores will be counted [17].
- Skeletal muscle mass and fat mass: The participants will be asked to dress in light clothing, hold the handgrips and stand still barefoot on a bioimpedance analyzer (Inbody 230, Biospace Co., Ltd, Seoul, Korea) after overnight fasting. The InBody 230 is a segmental impedance device which uses a tetrapolar 8-point tactile electrode method and two different frequencies (2 and 100 kHz). Fat mass, fat-free mass, percentage body fat and appendicular muscle mass (muscle mass of both arms and legs) are determined by the manufacturer’s algorithm of InBody 230. The appendicular skeletal muscle mass index (ASMI), expressed in kg/m^2^, is calculated by dividing the appendicular muscle mass by the square of height [41].

#### Secondary outcomes

The secondary outcomes evaluate older people’s experience of the exercise programmes. The measurements include exercise adherence, dropout rate, exercise maintenance, and Rosenberg Self-esteem Scale. Focus group interviews with open-ended questions will be conducted to evaluate the overall experience of older people with an emphasis on their psychological welfare, social well-being, and their acceptance of the telehealth approach.

- Exercise adherence will be reflected by the attendance rate which is the percentage of exercise sessions attended by the participants during the 3-month intervention period.
- Dropout rate: This will be assessed by the number of participants who refuse to continue the training during the intervention phase or refuse to provide information during the maintenance phase. The details of the dropout will be recorded.
- Exercise maintenance: This will be measured by the Physical Activity Scale for the Elderly (PASE), weekly activity level and exercise diary of the participants at the baseline, and after 3, 6 and 12 months. PASE is a widely used questionnaire in epidemiological studies for assessing the physical activity level of older people. It comprises 12 items addressing the leisure, physical, household and work-related activities over the past week [34]. Physical activity level will be measured objectively by accelerometers (ActiGraph GT3X, Pensacola, FL, USA). The participants will wear one on their waist for seven consecutive days at the baseline, and 3rd, 6th and 12th months. Their total steps, activity energy expenditure, metabolic equivalent of task scores and physical activity levels will be estimated by the ActiLife software.
- Rosenberg Self-Esteem Scale: This is one of the widely used measures of self-esteem. The scale includes 10 items rated on a 4-point scale ranging from 1 (strongly disagree) to 4 (strongly agree), with higher scores indicating higher self-esteem.

### Adverse event reporting

Any adverse events during the intervention and maintenance phase will be recorded. The details of any adverse event such as the type, the time and the severity, will be recorded and analyzed to ascertain whether it is related to the study. In the case of a serious adverse event during the class, we will review the causes, the prescribed exercises, the course structure, and the safety precautions. The concerned participant will be suspended from the study. The data analysis and reporting of such adverse events will follow the CONSORT statement for better reporting of harm-related data [42].

### Statistical analysis

Descriptive data of the two groups including age, risk of falls, body mass index, muscle mass, and activity level at the baseline will be analyzed by independent t-test to test the homogeneity of the physical characteristics between the two groups. We will use an intention-to-treat analysis based on all randomized participants following the guidelines on randomized controlled trials [43]. We will perform a sensitivity analysis for missing data. The Kolmogorov-Smirnov one-sample test will confirm the normal distribution of all the measurement variables. A two-way repeated measures ANOVA will be performed for all the outcome measures, accounting for the group (TE and CB) and the interaction term between group and time. The p-value will be set at 0.05 for all tests. All statistical analyses will be carried out using SPSS, version 26.0 (IBM Co., Armonk, NY) by an independent statistician who will not be involved in the outcome assessment. Missing data will be complemented by using the multiple imputation method.

Both groups of participants will also be invited to attend focus group interviews with an experienced qualitative researcher. The Theory Domain Framework will be adopted to examine the determinants of behaviour change [44]. Data analysis will follow a framework approach [45] and use Qualitative Data Analysis (QDA) Miner 5 of Provalis Prosuite, which is qualitative data analysis software.

### Data monitoring and quality control

The project assistant, who has a master’s degree in sports coaching and management and is a certified fitness coach, has solid experience delivering exercise classes to older people. He coordinates with the instructors, participants, social workers of the community centres and the other trained assistants. The instructor has a master’s degree in physical education and is a certified fitness instructor. He has over ten years of experience delivering exercise classes to older people and people with chronic diseases. Additionally, he already had one year of experience teaching exercise classes online before our study had started. The five trained assistants who will be blinded to the group assignment will perform assessments with the participants. The other ten trained assistants and the project assistant will arrange home visits for the participants of the TE group and set up the mobile device for the Zoom class. A family doctor with Specialist qualification in Community Medicine, an occupational therapist and an exercise physiologist will oversee the medical clearance and safety of the participants. The principal investigator and the project assistant will be responsible for monitoring the study and overseeing all the procedures.

### Data management and dissemination

Data will be collected by the outcome assessors using the tailored report forms. The project assistant will input the data into the computer and ensure their integrity and accuracy. The study protocol can be obtained through the Chinese Clinical Trial Registry Website (https://www.chictr.org.cn/hvshowprojectEN.html?id=219002&v=1.1) under registration number: ChiCTR2200063370 (Registered on 5 September 2022). The results of this study will be disseminated at an international conference, and by publications in high-impact peer-reviewed journals. The datasets generated during the current study will be available from the corresponding author upon reasonable request.

## Discussion

This study evaluates whether the tele-exercise approach is comparable to a traditional face-to-face exercise class in terms of physical functioning and exercise adherence during the 3-month intervention and the subsequent 9-month maintenance phase among the older people with possible sarcopenia or at risk of falls. Traditional face-to-face exercise classes allow the instructor to monitor older people’s performance and promote social interaction. However, the time and space available for such group classes for older people are limited. Frail older people may have less confidence to leave their home to attend classes in the community [8]. They may need support from their family. The transportation arrangements may also affect how likely they will participate in the programme. The recent social distancing policy during the COVID-19 pandemic has also been a crucial factor resulting in the frail older people baulking from participation in exercise programmes in the community. Consequently, health professionals are also urging governments to prepare for the next pandemic [46]. Considering older people are more willing to do strength and balance training at home [47], a feasible and effective alternative should be offered to them. The tele-exercise approach in this study uses an online communication application, Zoom, to deliver live group exercise classes in their home. It is convenient in that this approach avoids commuting time and older people can attend classes even in bad weather and could also do so during the pandemic. Frail older people can exercise with others at the same time with less social consciousness and peer pressure [48]. The strength of the current study is that we consider the needs of older people to interact and their fear of new technology. Each lesson will be well structured with a short break every 10 min to allow interaction in both groups. The team will arrange a home visit to the TE group before the intervention period and guide the setting up of the tablet and their home environment such as the angle of the tablet stand, where the tablet and chair should be positioned and where the participant should stand. During the lessons, our team will control the tablet of each participant remotely to minimize any technological problems that they may encounter.

Regular physical activities are well-known to have benefits in regard to balance, muscular strength and functional mobility particularly for older people. The benefits depend on the adherence to their exercise regimen and the satisfaction that they derive from it. Older people with a higher perceived risk of falling are associated with poorer exercise adherence and their adherence to exercise interventions decreases over time [49]. The main barriers include environmental constraints, resources (cost and aversion to gyms), behavioural regulation (motivation) and emotion (boredom) [50]. In other words, if older people are motivated and encouraged continuously, they can exercise in an environment in which they feel comfortable, and if the exercise programme is interesting without much cost, older people are more likely to maintain their exercise habit. The current study aims to address the physical functioning of older people and their experience with an exercise programme. Therefore, strategies to enhance their exercise experience are emphasized based on the findings of previous studies as described under the “Methods” section. The areas we will focus on are namely self-efficacy, self-confidence, and empowerment [17, 49]. The instructor and the speakers for health talks will also be older indivduals who will serve as a role model to enhance the confidence of the participants in maintaining regular exercising habits and adopting a healthier lifestyle. The modified Otago exercise programme is adopted in this study as it has been shown to be an effective home tailored exercise programme to prevent falls among older people [24]. The problems of lack of engagement and interesting variations of movements of the original OEP will also be addressed. The lesson plan will emphasize interaction by allowing short breaks between sessions. The instructor and the project assistant will approach the participants to elicit their comments. The last session of each lesson will be “Workouts on-demand”. Additional exercises such as those focused on mobility, flexibility and strength training for various body parts will be introduced based on the comments of the participants in addition to the OEP.

The major concern of the research team is the safety of the older people at risk of falls while exercising at home alone. All the participants will have met the inclusion criteria and pass the preparticipation Health Screening of the American College of Sports Medicine (ACSM). Potential participants diagnosed with dementia or cognitive impairment and severe vision impairments will be excluded from the study. We have a strong team to uphold the safety of the programme. A family doctor is responsible for the medical clearance of the participants. The instructor and the project assistant are experienced in delivering exercise classes to older people. They have familiarized themselves with the safety protocol and understand when to call for emergency help. The OEP adopted in the current study is well-known for its safety. Even for unsupervised home-based OEP, two studies showed that none of the participants who were older fallers with a mean age ranging from 76.2 to 81.4 years reported any symptoms or adverse events associated with the exercises [51, 52]. In another study [53], two participants reported lower back pain associated with the exercises. One resumed exercising and the other discontinued the exercises. The rate of adverse events associated with the OEP is relatively low. A systematic review conducted by Mañas et al. [54] evaluated the safety issues of unsupervised home-based resistance training for community-dwelling older adults in 21 studies and reported no major adverse events. It was also found that the incidence of adverse events was similar between the training intervention group and the control groups, suggesting that unsupervised home-based resistance training is safe [54].

The study has three main limitations. Firstly, due to budget constraints, we can only lend equipment to participants of TE during the intervention period. Online exercise courses offered by the local course providers commonly require participants to have their own devices and Wi-Fi connection, with minimal technical support provided. Participants who borrowed tablets and sim cards from us may not be able to continue their exercise online during the maintenance phase. Instead, we suggest that all participants, including both TE and CB, aim to achieve 10,000 steps daily. They should also follow the guidelines in our exercise booklet and exercise videos, which outlines our exercise programme for practicing at home. Furthermore, we encourage participants to take advantage of exercise courses available in their district. Secondly, as travel restrictions are relaxed after the COVID-19 pandemic, some participants may plan to stay outside of Hong Kong for an extended period, which could impact their ability to maintain their activity level during the maintenance phase. Thirdly, the administration of a large number of tests at different time points may increase the dropout rate and lead to missing data. Therefore, it is important to pay greater attention to these areas and implement strategies to minimize these problems. Possible strategies could include regular phone calls and text messages to the participants, well-trained outcome assessors and regular monitoring of the data collection procedures by the project assistant.

This paper presents the design of a clinical trial to examine the possibility of a tele-exercise approach for the older people with possible sarcopenia or at risk of falls. The intervention of the current study includes 3 months of supervised exercise delivered either online (TE) or face to face (CB) which helps the participants to gain confidence to build up their exercise habits during the subsequent maintenance phase. The team has focused intensively on the different strategies to enhance the exercise experience of the participants and their safety. The comprehensive assessments of the study can help to propose an exercise delivery and maintenance model for future practices.

### Trial Status

Protocol version number: 1.1.

Date of recruitment began: September 2022.

Recruitment will be completed when 92 participants are enrolled.

Current status: 2 groups of CB completed 3 month intervention. 1 group of CB and 2 groups of TE are in the intervention phase. We will recruit 5 more groups to meet the target.

## Data Availability

Data sharing is not applicable to this article as no datasets were generated or analysed during the current study.
